# The effect of problem-solving and assertiveness training on self-esteem and mental health of female adolescents: a randomized clinical trial

**DOI:** 10.1186/s40359-023-01154-x

**Published:** 2023-04-09

**Authors:** Parastoo Golshiri, Alireza Mostofi, Shiva Rouzbahani

**Affiliations:** Department of Community Medicine and Family Physician, School of Medicine, Isfahan University of Medical Sciences, Isfahan, Iran

**Keywords:** Problem-solving skill, Assertiveness, Self-esteem, Mental health, Female, Student, Adolescent

## Abstract

**Background:**

Children and adolescents are the assets of the society and their mental and physical health ensures the future health of next generations. The aim of the current study was to evaluate the effect of problem-solving and assertiveness skill training on improving self-esteem and mental health in high school female students of Isfahan city in 2019.

**Methods:**

This study was a randomized clinical trial. The target population was 10th grade female high school students in Isfahan city of Iran. 96 students of a female public high school were included in  the study, allocated to two groups, 32 for intervention group and 64 for control group. Six 90-min sessions were considered for problem-solving and assertiveness skill training which included lectures, question and answer, movie displaying, brainstorming and role-play. The Coopersmith Self-esteem Inventory Questionnaire (CSEI) and General Health Questionnaire (GHQ) were used in order to evaluate the variables of the study before and one month after the intervention.

**Results:**

Mean scores of the self-esteem variable changed significantly in the intervention group compared to control group before (25.2 ± 2.905) and after (29.9 ± 4.155) the intervention (*p* < 0.05). Mean scores of mental health also changed significantly before (27.67 ± 5.42) and after (19.033 ± 4.9) the intervention in comparison with the control group (*p *< 0.05).

**Conclusion:**

The findings of the present study showed that educational intervention based on problem-solving and assertiveness can  enhance self-esteem and mental health in students. Future studies are needed to confirm and determine the structure of these associations.

*Trial Registration* IRCT Code: IRCT20171230038142N9. Registration Date: 07/07/2019. Ethics Code: IR.MUI.MED.REC.1398.130.

## Background

Childhood and adolescence are among the most important stages of life. Children and adolescents are very important parts of the society and their mental and physical health ensures the future health of the society. Adolescents’ lives are accompanied by special difficulties and complications such as social and familial transformations, dangerous disease incidence, competition, exam stress, etc. Each one alone can bring on a lot of tension and put their health in risk which could lead to irreparable damage. According to this, knowledge of life skills and problem solving affects different aspects of students’ behavior and personality. Nowadays many researchers believe that coping skills such as problem-solving skill, providence and making reasonable and accurate assessment helps people cope with problems more successfully [1].

Another one of these important skills is the problem-solving skill which is a necessity to obtain behavioral and social health and to have a generally successful performance in life. The results of different studies show that assertiveness skill training improves mental health. On the other hand, self-esteem and self-efficacy are some of the most essential factors for optimal growth of children and adolescents’ personality. Self-esteem is considered to be one of the most important components of mental health as the central and basic factor in social-emotional adjustment [2].

There are some studies that have evaluated the epidemiological status of mental disorders in different communities. In a study on epidemiology of depression and anxiety among 1582 undergraduate students in 2021, the prevalence of depression and anxiety turned out to be 22.3% and 15.8%, respectively. This study indicated that girls were more likely to show such symptoms [3].

In a WHO World Mental Health Surveys International College Student Project, 35% of the samples among 13.984 students from 19 colleges across 8 countries demonstrated positive screening for one or more of the common lifetime disorders [4].

The study of Hashemian and his colleagues in Isfahan estimated the prevalence of depression in students by 29.1 (32.9 in girls and 25.4 in boys) [5].

Nathan King and colleagues’ work showed higher possibilities of anxiety and depressive diagnosis in females [6].

Suicide is one of the preventable outcomes of mental health disorders worldwide caused by different factors. Zarei and his colleagues’ evaluation on factors associated with suicidal attempts demonstrated that stressful life events and perceived social support are two of the most important factors in attempting suicide [7].

According to a meta-analysis and systematic review conducted in 2019 including 24 studies and 25.354 participants, suicide plays the second role in young people’s death [8].

Another meta-analysis study among 37.486 undergraduates in 2021 estimated the pooled prevalence of depression, anxiety and suicidal behaviors to be 28.51%, 37.75% and 9.10%, respectively [9].

It appears that problem-solving and assertiveness training could play a major role in improving their communication skills [10]. Regarding the fact that today’s girls could be future’s mothers of society and the prevalence of mental disorders is higher among girls in comparison with boys, especially in Iran [11], also due to the importance of effective educational methods for life skills training in order to promote mental health in the adolescence age group, researchers decided to develop an interventional program with problem-solving and assertiveness skill training for female adolescents in order to evaluate its effect on their self-esteem and mental health.

## Methods

**Fig. 1:**
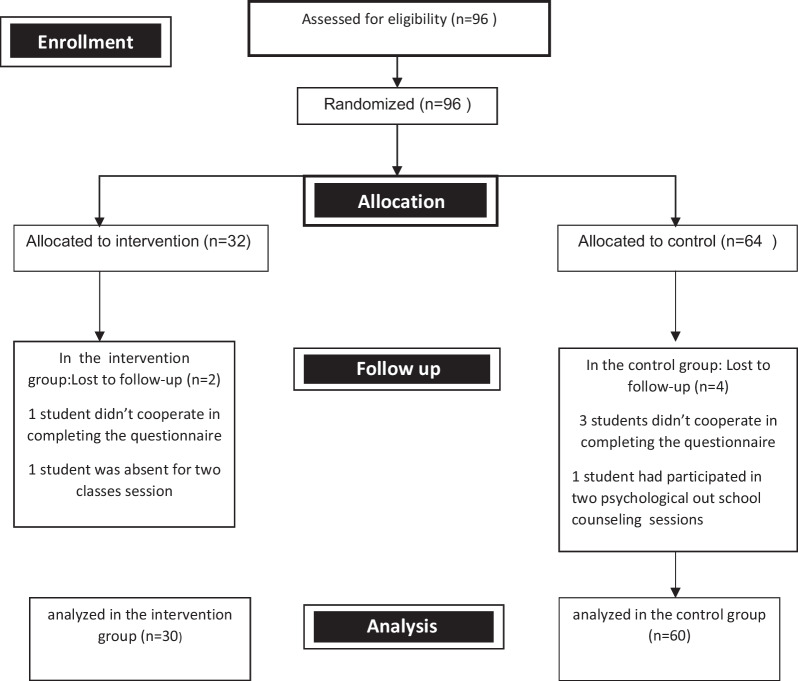
Research flow-diagram (Based on Consort 2010 flow diagram. http://www.consort-statement.org/consort-statement/flow-diagram)

This study was a randomized clinical trial. The target population was 10th grade female high school students in the age group of 15–16 years old in Isfahan city in the center of Iran in 2019. There are two types of schools in Iran, public and non-profit schools.The students in public schools  usually live in the same neighborhood as the school is located.Since the researchers wanted to choose a public school for girls from an area with moderate socioeconomic status in the city of Isfahan, by referring to the Regional Organization of Education, 3 school were introduced to us with the  same characteristics we wanted. Among these three schools, we randomly selected one. In this school, 96 students were studying in the 10th grade. All students were included in the study by census sampling. In order to perform random allocation, first the researchers received the student numbers of the 10th grade students from the school's executive director and then entered these numbers into the SPSS software, and then using this software, the students were allocated in two groups of 32 and 64 people (32 in intervention group and 64 in the control group). The researchers reported the numbers extracted from the software to the executive director of the school and thus, the names of the students in each group were determined. ( Flow diagram of the study was given in Fig. 1).

The inclusion criteria were informed consent to participate in the study by the students and their parents. Exclusion criteria were as follows:

1. Participation in other psychological programs during the intervention course.
2. History of psychological medicine use or any mental disorders.
3. Absence for at least two sessions of training classes for the intervention group.

The place where educational sessions were held and assessed was the school hall. There was no stopping guidelines for the protocol in this research. Since designed trainings were provided  in the intervention group, and the control group only received routine school training, and also both groups were in the same school, there was no possibility of blinding the participants. Only the person who entered the data and performed the statistical analysis was unaware of the studied groups.

Six 90-min sessions were considered for problem-solving and assertiveness skill training which included lectures, question and answer, movie displaying, brainstorming and role-play. The role-plays were accompanied by rehearsals at home and assignments for the next session. In the second session, the students were given a pamphlet about life skills for further study. At the end of every session, homework was assigned based on the topics discussed. Reviewing the assignments at the beginning of every session, answering the question and giving feedback were also a part of each session. Empathy was used as one of the most important factors of communication with students in all sessions. The content of the sessions (shown in Table 1), was compiled based on WHO protocols [18]. No intervention was considered for the control group and they were only in the current programs of school.

**Table1:** Teaching methods and contents of every session

Sessions	Actions, methods, techniques and contents
First session	Explaining the goals of taking part in the group, displaying media content affective on motivation of the target group, using simple and creative methods like preparing a notebook for solving problems from today until the rest of their lives (for creative naming and problem-solving) Using balloons and writing down fears on them, then bursting them in order to eliminate the fear Stablishing sincere relationships by involving class members in expressing their dreams and goals
Second session	Enhancing students’ self-confidence by hanging motivational sentences in the classroom Attempting to change the mental model of members from “I cannot” to “I can” using age-appropriate stories Defining assertive and unassertive behavior by describing and comparing it using two different stories Practicing critical thinking and expressing opinions using plays designed by students
Third session	Practicing assertiveness by involving each of the class members in discussions Teaching problem defining as the next step in problem-solving skill Teaching body language using role-play and explaining its importance in a simple way Explaining individual rights and using visual contents in the class Considering anger management as a method of reaching a suitable solution to the problems ahead
Forth session	Teaching brainstorming method as the next step in problem-solving and practicing it in class Promoting self-confidence by emphasizing on satisfaction with individual differences and physical features by telling informative stories Teaching how to understand individual rights and express opinions by involving participants in discussions
Fifth session	Using positive imagination and storytelling for motivation Teaching If–Then technique as the next step in problem-solving process and using decision-making tables technique Rebuilding the issues that they have had encountered with at home and at school during these sessions by role-playing Practicing how to reject unreasonable requests by expressing students’ experiences in the classroom Accepting individual and physical differences and expressing dreams in public as a technique to enhance assertiveness
Sixth session	Emphasizing on consulting in order to reach the right solution to problems Managing stress as an important issue and by mentioned methods, and brainstorming solutions designed by students Saying goodbye and encouraging students to continue the practices by hanging the sentence “I should learn the skill of problem-solving and assertiveness in order to reach my dreams” on the class board

Two questionnaires (CSEI questionnaire and GHQ 28) were completed by the students.Before starting the interventions, the students were given the questionnaires and the researchers helped the students complete them, and their questions were answered. After one month from the beginning of the intervention, the questionnaires were completed again by the students under the supervision of the researchers.

1. The Coopersmith Self-Esteem Inventory (CSEI): We used the CSEI questionnaire (1967) in order to assess the self-esteem of the participants. This questionnaire has been widely used in several studies. Form A (the school form for adolescents age 8 to 16 years) consists of 58 items which eight of them are lie detectors. If the person scores more than four from those eight lie scale items, it means that the validity of the test is low and they have attempted to make themselves look better than they really are. The scoring method of this test is zero—one. The total 50 items are divided into four scales, representing different aspects of self-esteem including general self, social self-Peers, home parents and school academic. These scales, in addition to four other subscales, yield an overall score. The minimum score of general-self subscale will be zero with a maximum score of 26. The other three subscales will have a minimum score of zero and a maximum of eight. The maximum score possibly obtained is obviously 50 and the minimum is zero, and higher scores represent higher self-esteem levels. According to the scoring of this questionnaire, a total score of 26 or less indicates poor self-esteem, between 27 to 34 marks average and above 44 is considered strong self-esteem [12]. Several studies have pointed out the high validity and reliability of the CSEI questionnaire. Mahdavi and his colleagues (2010) had already translated this questionnaire into participants’ native language and evaluated the validity and reliability of it. They used α Cronbach’s method in their research to determine the reliability of the questionnaire which turned out to be 0.60 [13].
2. General Health Questionnaire (GHQ 28): The main form of the questionnaire was developed by Goldberg and Hillier in 1970 and its reliability and validity have been investigated many times since then [14]. In simultaneous evaluating the validity of this questionnaire with Minnesota multilateral questionnaire, Chan and Chan reported a 0.54 correlation coefficient [15]. Kalman and Wilson also reported a 0.69 concurrent validity coefficient while assessing this questionnaire with frustration scale. The sensitivity of GHQ28 is 0.84 and its specificity is 0.82 [16]. In their research on the psychosomatic properties of GHQ in Iranian population, Ebrahimi and colleagues obtained a clinical cut off point, sensitivity specificity, and general classification error of 24, 0.80, 0.00 and 0.1, respectively. They also obtained criterion validity Cronbach’s alpha and split reliability co-efficient to be 0.78, 0.97 and 0.90, respectively [17]. According to the scoring of this questionnaire, a total score of less than 21 indicates a good mental health status, between 22 and 42 indicates that in some areas the person is exposed to threats and injuries, and above 43 indicates a serious condition more in terms of mental health. In each component, a score of 0 to 9 indicates a good condition, a score of 10 to 15 indicates a moderate condition, and between 16 to 21 indicates a worse condition [14].

The GHQ questionnaire divides into four subscales as follows:

1. Somatic symptoms: Including items about how people feel about their health, feeling tired along with physical symptoms (Question 1–7 in GHQ 28).
2. Anxiety/Insomnia: Including items related to insomnia and anxiety (Q 8–14 in GHQ 28).
3. Social dysfunction: Evaluates people’s ability to cope with the demands and words and issues of daily life and reveals their feelings about coping with common life situations (Q 15–21 in GHQ 28).
4. Severe depression: Including severe depression cases and suicidal tendencies (Q 22–28 in GHQ 28).

All the parents of the students and the students themselves agreed to participate in the study and signed the consent form. During the intervention, 2 students were excluded from intervention group and 4 students were excluded from control group (shown in flow diagram of the study).

In order to observe ethics in the research, a summary of the training program in the intervention group with pamphlets were also given to the control group after three months of intervention.

The data was analysed using SPSS version 16.0. Chicago, SPSS Inc., Independent *T*-test and paired *T*-test were used in the analysis of self-esteem and mental health. *P* < 0.05 was considered significant. Based on the ethics rules of the school, we didn’t have permission to get any other demographic information. Therefor, we didn’t perform additional analyses, such as subgroup analyses and adjusted analyses.

## Results

From 96 10th grade female students in the age group of 15–16 years old who entered the study, 90 were analyzed finally in this research( 30 of them were in the intervention group and 60 were in control group). We compared these groups in the inferential data part by comparing means and using standard deviation. Results of The Coopersmith Self-Esteem Inventory (CSEI) showed that mean scores of general-self (*p* = 0.03), home-parents (*p* < 0.001), social self-peers (*p* < 0.001) and school academic(*p* = 0.04) significantly increased in the intervention group in compare with the control, so the intervention had a positive effect on the four components in the field of self-esteem in students. Finally, the total scores of self-esteems significantly increased in the intervention group (*p* < 0.001) (Table 2).

**Table 2:** Comparison of the mean of the two groups and within each group about the self-esteem variable

Component	Group	Before intervention M ± SD	After intervention M ± SD	*P*2
General self	Control (*n* = 60)	13.46 ± 2.39	13.53 ± 1.93	0.74
	Intervention(*n* = 30)	13.10 ± 2.00	14.43 ± 1.67	< 0.001
	P1	0.48	0.03	
Home-parent	Control	3.68 ± 1.32	3.87 ± 1.10	0.32
	Intervention	4.26 ± 1.41	5.43 ± 1.10	< 0.001
	P1	0.06	< 0.001	
Social self-peer	Control	3.83 ± 1.40	3.88 ± 1.27	0.85
	Intervention	3.76 ± 1.83	4.93 ± 1.17	< 0.001
	P1	0.84	< 0.001	
School academic	Control	4.16 ± 1.42	4.06 ± 1.03	0.67
	Intervention	3.80 ± 1.06	4.56 ± 1.10	< 0.001
	P1	0.21	0.04	
Total score	Control	25.01 ± 3.23	25.25 ± + 2.89	0.69
	Intervention	24.79 ± 2.47	29.36 ± 2.39	< 0.001
	P1	0.75	< 0.001	

*P*1: Independent *T* test (at level of 95% confidence interval)

*P*2: Paired *T* test (at level of 95% confidence interval)

Also, in the intervention group, mean scores of four components of self-esteem were significantly increased after intervention (all *p* < 0.001).

Considering the total score of students' self-esteems in the control group after the intervention (25.25), it seems that according to the test interpretation, these students have poor self-esteem, but in the intervention group, this score has increased to 29.36, indicates an increase in self-esteem to a moderate level.

Results of General Health Questionnaire (GHQ 28) showed that mean scores of Somatic Symptoms (*P* = 0.002), Anxiety/Insomnia (*p* = 0.04), Social Dysfunction (*p* = 0.002) and Severe Depression (*p* = 0.04) significantly decreased in the intervention group in comparison with the control, so the intervention had a positive effect on the mental health status of the students. (Table 3).

**Table 3:** Comparison of the mean of the two groups and within each group about the mental health variable

Component	Group	Before intervention M ± SD	After intervention M ± SD	*P*2
Somatic symptoms	Control(n = 60)	3.45 ± 1.45	3.40 ± 1.39	0.60
	Intervention(n = 30)	3.63 ± 1.27	2.56 ± 0.72	< 0.001
	P1	0.55	0.002	
Anxiety/insomnia	Control	3.50 ± 2.09	3.80 ± 1.63	0.06
	intervention	3.73 ± 1.25	3.13 ± 1.00	0.004
	P1	0.57	0.04	
Social dysfunction	Control	6.36 ± 2.33	6.10 ± 1.81	0.07
	intervention	6.80 ± 2.41	4.90 ± 1.47	< 0.001
	P1	0.41	0.002	
Severe depression	Control	3.05 ± 1.69	3.23 ± 1.39	0.09
	intervention	3.23 ± 0.67	2.70 ± 0.46	< 0.001
	P1	0.57	0.04	
Total score	Control	16.36 ± 4.13	16.53 ± + 3.10	0.56
	intervention	17.40 ± 2.55	13.30 ± 1.98	< 0.001
	P1	0.21	< 0.001	

*P*1: Independent T test (at level of 95% confidence interval)

*P*2: Paired T test (at level of 95% confidence interval)

According to the total score of the students in this questionnaire and based on the interpretation of the results of this test, it seems that the mental health status of students in both groups has been favorable, but after the intervention, the mental health status of the control group has improved significantly (*p* < 0.001).

## Discussion

This study aimed to evaluate the effect of problem-solving and assertiveness skill training on self-esteem and mental health improvement of 10th grade female student. The statistical analysis results demonstrated a significant difference in both self-esteem and mental health variables between intervention and control groups. Also, all self-esteem subscales such as general self, home parents, social self-peers and school academic showed significant statistical difference before and after the intervention. Significant difference in all four subscales of mental health variable including somatic symptoms, social dysfunction, anxiety/insomnia, and severe depression was obvious before and after the intervention, as well.

In the following, there are examples of some studies similar to our findings. Adawi and colleagues (2016) showed that problem-solving skills have a positive significant effect on creativity and self-expression of students [19]. The study of Abootorabi Kashani and his colleagues (2012) also signifies that life skills training with more emphasize on assertiveness skill increases self-esteem of 9–11-year-old female students [20]. Mousavizadeh and colleagues compared effectiveness of problem-solving and assertiveness skill training on marital satisfaction rate of Allameh Tabatab’i University female students in 2012. The results showed that both methods are equally effective [21]. In Jafarigiv and Peyman’s study in 2019 on the effect of life skill training using self-esteem and self-efficacy literacy strategies, participants’ scores of both self-esteem and self-efficacy were significantly improved after the intervention [22]. The study of Xia and colleagues showed that positive family atmosphere and efficient parenting leads to better problem-solving skills and less violent behaviors among adolescents. Their findings indicated a positive association between assertiveness and problem-solving skills among young people [23]. There are other studies in adolescents’ mental health field which clarifies that other factors such as emotional intelligence can affect psychological components in this age group [24]. In this regard, in 2009 Chung and Chiou showed that mental health is under the effect of many variables such as quality of life and gender [25]. A longitudinal study from China also confirmed gender differences in mental disorders among college students. Anxiety was more common in female students [26] which was similar to our country [11].

To explain such improvement in variables, it is important to remember that problem solving is one of the constructive skills which can directly affect people’s mental health. Experts consider lack of self-esteem and reduced mental health in different conditions as one of the most important struggles. They believe that if people respond immediately while facing a problem, there may not be enough time for cognitive responses. Therefore, problem solving method can be effective in controlling the emotional reaction and this could be how problem-solving skill training may have a positive effect on self-esteem [21].

On the other hand, there are other studies that show a significantly positive correlation between self-esteem and mental health in which more self-esteem comes with higher levels of mental health [27]. Assertiveness skill leads to closer and more intimate relationship with people. It appears that students need to learn more social skills in school in order to value their own personal space and desires while respecting others’. They can be taught to express their desires and opinions and to show their feelings in an effective positive way. Thus, it is safe to say that training can increase mental health and self-esteem [21].

### Limitations of the study

Based on the ethics rules of the school, we didn’t have permission to get any other demographic information. Other demographic information such as economical condition or parent situation may have had some effects on the outcomes and could have been modified in data analysis. Since the students of one school participated in this research, it was not possible to separate the intervention group and the control group during the implementation of the project. Therefor, it could have been possible that some trainings were transferred between the two groups by the students of the intervention group. It is also important to consider that generalizing the results had to be done with caution since this study was conducted in a specific age group and female gender.

## Conclusion

Based on the results of the present study, it can be concluded that educating students on life skills such as problem-solving and assertiveness skill in school, in addition to teaching routine lessons, can be highly effective in their self esteem and mental health improvement. All self-esteem subscales such as general self, home parents, social self-peers and school academic have improved after the intervention. The subscales of the mental health variables also showed significant changes. It seems that students need to learn more life skills in school in order to promote empowerment and become more capable in personal and social life. Thus, it is safe to say that training life skills is one the basic needs for children which should be considered in addition to conventional education in schools.

## Data Availability

The datasets used and/or analyzed during the current study are available from the corresponding author on reasonable request.

## Abbreviations

WHO: World Health Organization
CSEI: Coopersmith self-esteem inventory
GHQ: General health questionnaire
